# Proteasomal degradation of the histone acetyl transferase p300 contributes to beta-cell injury in a diabetes environment

**DOI:** 10.1038/s41419-018-0603-0

**Published:** 2018-05-22

**Authors:** Lucie Ruiz, Tatyana Gurlo, Magalie A. Ravier, Anne Wojtusciszyn, Julia Mathieu, Matthew R. Brown, Christophe Broca, Gyslaine Bertrand, Peter C. Butler, Aleksey V. Matveyenko, Stéphane Dalle, Safia Costes

**Affiliations:** 1 IGF, CNRS, INSERM, University of Montpellier, Montpellier, France; 2 Larry L. Hillblom Islet Research Center, David Geffen School of Medicine, University of California Los Angeles, Los Angeles, CA USA; 3 Laboratory of Cell Therapy for Diabetes (LTCD), Institute for Regenerative Medicine and Biotherapy (IRMB), University Hospital of Montpellier, Montpellier, France; 4 Department of Endocrinology, Diabetes, and Nutrition, University Hospital of Montpellier, Montpellier, France; 5 Department of Physiology and Biomedical Engineering, Mayo Clinic School of Medicine, Mayo Clinic, Rochester, MN USA

## Abstract

In type 2 diabetes, amyloid oligomers, chronic hyperglycemia, lipotoxicity, and pro-inflammatory cytokines are detrimental to beta-cells, causing apoptosis and impaired insulin secretion. The histone acetyl transferase p300, involved in remodeling of chromatin structure by epigenetic mechanisms, is a key ubiquitous activator of the transcriptional machinery. In this study, we report that loss of p300 acetyl transferase activity and expression leads to beta-cell apoptosis, and most importantly, that stress situations known to be associated with diabetes alter p300 levels and functional integrity. We found that proteasomal degradation is the mechanism subserving p300 loss in beta-cells exposed to hyperglycemia or pro-inflammatory cytokines. We also report that melatonin, a hormone produced in the pineal gland and known to play key roles in beta-cell health, preserves p300 levels altered by these toxic conditions. Collectively, these data imply an important role for p300 in the pathophysiology of diabetes.

## Introduction

Pancreatic beta-cells synthesize and secrete insulin, the key regulatory hormone of glucose metabolism through its action to constrain hepatic glucose production and stimulate glucose uptake in skeletal muscle and fat. Type 2 diabetes (T2D) is a metabolic disorder characterized by a progressive deterioration of beta-cell mass and function in the setting of insulin resistance. The beta-cell deficit and beta-cell failure in T2D are likely related to beta-cell stress and apoptosis^1, 2^ in response to a variety of stress factors including amyloid deposits, chronic hyperglycemia and hyperlipidemia, and/or low grade-inflammation. The preservation of a functional beta-cell mass is essential to maintain glucose homeostasis. Beta-cell function and survival are controlled by fine regulation of gene expression in response to physiological stimuli and metabolic changes. Among the mechanisms involved in gene regulation, remodeling of chromatin structure by epigenetic mechanisms is a fundamental process. Histone acetylation is a regulatory mechanism capable of modulating properties of chromatin and thus the competence of the DNA template for transcriptional activation. Histone acetylation is catalyzed by the chromatin-modifying enzymes lysine/histone acetyl transferases (HATs)^3^ and the reversed deacetylation process by lysine/histone deacetylases (KDACs or HDACs)^4^. Whereas accumulating evidence suggests the importance of KDACs for the maintenance of beta-cell function and survival^5–7^ (for review, see Campbell et al.^8^), roles of HATs in beta-cells and their alteration under pathophysiological conditions remains little investigated.

Among the HAT family members, the co-activator p300 is a key component of the transcriptional machinery involved in diverse biological processes, including differentiation, development, proliferation^9^, and circadian function^10^, but also in numerous pathophysiological processes, including several forms of cancers and cardiac hypertrophy^11, 12^.

In beta-cells, p300 is recruited to the insulin gene promoter in response to glucose via its interaction with the transcription factors PDX-1^13^, Beta-2, and E47^14^. P300 also regulates PDX-1 transcription in beta-cells via its interaction with the Maturity Onset Diabetes of the Young (MODY)-associated transcription factor KLF11^15^. In patients with T2D carrying mutations for Beta-2/NeuroD^16^ and PDX-1^17^, the ability of beta-cells to produce sufficient amount of insulin is compromised. Interestingly, mutations of these genes precisely affect the p300-interacting domain^16, 18, 19^, suggesting that a defect in p300 could be a cause for beta-cell dysfunction. Recently, a computational analysis identified some T2D-associated single nucleotide polymorphisms (SNPs) that were located at transcription factor binding sites including p300 (*EP300*)^20^, further suggesting a potential involvement of p300 in the pathophysiology of T2D.

Whereas p300 appears as a central integrator of various signaling pathways, the regulation and biological actions of p300 in pancreatic beta-cells remain elusive. Here, we sought to study the potential role of p300 in beta-cell survival and to investigate its mechanism of regulation in beta-cells exposed to stress situations known to be associated with T2D.

## Results

### Loss of p300 acetyl transferase activity and expression leads to beta-cell apoptosis

**Fig. 1:**
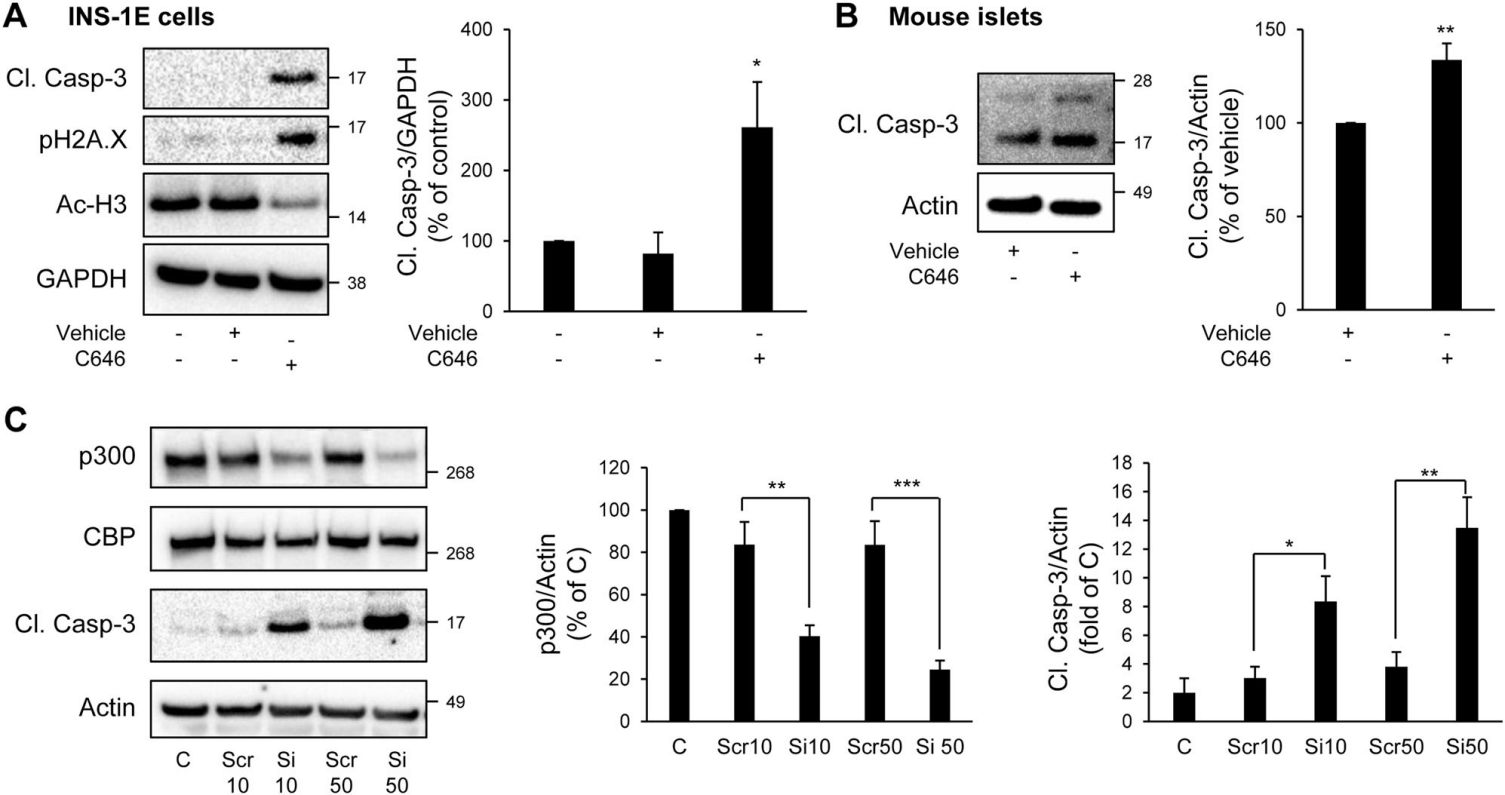
Inhibition or knock-down of p300 leads to beta-cell apoptosis. **a** INS-1E cells were treated with C646 (30 μM for 24 h) (or with 0.003% DMSO as vehicle). Levels of cleaved caspase-3 (Cl. Casp-3), Phospho-Histone H2A.X (Ser139) (pH2A.X) and acetyl-Histone H3 (Ac-H3) were assessed by western blot. GAPDH was used as loading control. The graph represents the quantification of the western blot (*n* = 4). **b** Isolated mouse islets were treated or not with C646 (30 μM for 48 h). Levels of cleaved caspase-3 (Cl. Casp-3) were assessed by western blot. Actin was used as loading control. The graph represents the quantification of the western blot (*n* = 3). **c** INS-1E cells were transfected with scramble (Scr) or p300 siRNA (Si) (10 or 50 nM) during 48 h; (**C**, non-transfected cells). p300, CBP and cleaved caspase-3 (Cl. Casp-3) protein levels were analyzed by western blot. Actin was used as loading control. The graphs represent the quantification of p300 and cleaved caspase-3 protein levels (*n* = 4). Data are expressed as mean ± SEM; *P < 0.05, **P < 0.01, ***P < 0.001

To evaluate whether p300 loss could play a role in vulnerability of beta-cells to apoptosis, we treated INS-1E cells with C646, a selective, potent and cell-permeable inhibitor of p300 acetyl transferase activity^21^. As a control of its efficacy, acetylation levels of Histones H3, targets of p300, were diminished by 43% in C646-treated INS-1E cells (*P* < 0.05; Fig. 1a). Inhibition of p300 acetyl transferase activity in INS-1E cells led to increased caspase-3 cleavage and histone H2AX phosphorylation, a marker of beta-cell apoptosis^22^, and of beta-cell death-associated DNA fragmentation^23^, respectively (Fig. 1a). In mouse islets treated with C646, an increase in caspase-3 cleavage was also clearly detected (Fig. 1b). To further confirm the observation that p300 is important for beta-cell survival in primary cells, we evaluated the frequency of TUNEL-positive beta-cells in both isolated mouse and human islets treated with C646. The frequency of TUNEL staining in mouse beta-cells was increased by 2.7-fold in islets treated with C646 (*P* < 0.05; Supplemental Fig. 1A and B), whereas the frequency of TUNEL staining in alpha-cells was not significantly different (Supplemental Fig. 1B). Similarly, the frequency of TUNEL staining in human beta-cells was increased by 1.6-fold under C646 treatment (Supplemental Fig. 1C and D). In addition, inhibition of p300 by C646 also led to an altered beta-cell function, as shown by the decreased expression of the transcription factors Pdx1 and Nkx6.1 (Supplemental Fig. 2A) and the decreased insulin stimulation index (Supplemental Fig. 2B and C)

To further ascertain the involvement of p300 in beta-cell survival and function, we used a siRNA approach to specifically target p300 and decrease its expression. Transfection of INS-1E cells using 10 nM and 50 nM of siRNA for 48 h resulted in 51.6 ± 5% and 72.4 ± 3.7% knockdown of p300 protein content, respectively (Fig. 1c). The decrease in p300 protein content was associated with a decrease in p300 activity, as shown by decreased acetylation levels of Histones H3 (Supplemental Fig. 3). This decrease in p300 protein content and activity resulted in increased beta-cell apoptosis illustrated by the cleavage of caspase-3 (Fig. 1c), but also in an alteration of beta-cell function, as shown by the decreased insulin stimulation index (Supplemental Fig. 4). In conclusion, both p300 inhibition and invalidation data reveal a novel role for p300 in beta-cell function and survival.

### Diabetes-related conditions induce a loss in p300 protein levels in beta-cells

**Fig. 2:**
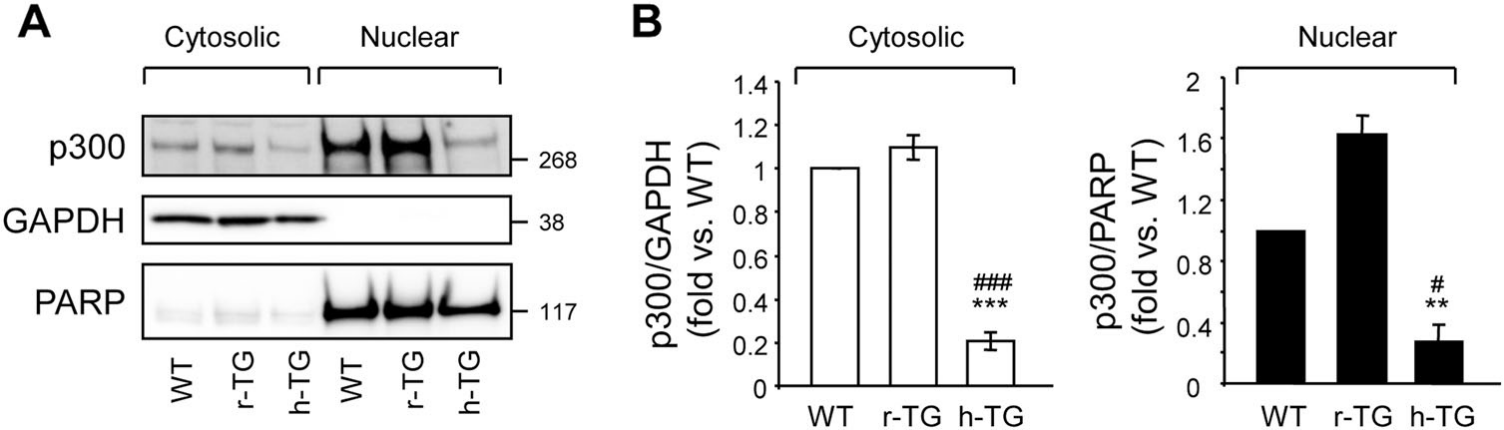
Increased expression of h-IAPP decreases cytosolic and nuclear protein levels of p300 in mouse islets. **a** Islets isolated from 9 to 10 week-old WT (wild type, *n* = 6), r-TG (rodent-IAPP transgenic, *n* = 6), h-TG (pre-diabetic human-IAPP transgenic, *n* = 4) mice were subjected to subcellular fractionation. Cytosolic and nuclear fractions resolved by SDS–PAGE and immunoblotted with anti-p300, anti-GAPDH (loading control for cytosolic fraction), anti-PARP antibody (loading control for nuclear fraction). **b** Quantification of p300 protein levels (*n* = 3). Data are expressed as mean ± SEM.; ^#^*P* < 0.05 and ^###^*P* < 0.001 vs WT

We next examined whether p300 levels could be modulated in several conditions known to be associated with T2D. The islets in T2D are characterized by the presence of toxic oligomers of human islet amyloid polypeptide (h-IAPP)^24^. Transgenic expression of h-IAPP in mouse islets (h-TG mice) leads to development of diabetes with an islet pathology that recapitulates features of beta-cell demise in human T2D^25^. We examined islets of mice with comparable expression of the oligomeric human form of IAPP (h-TG) versus the soluble rodent form of IAPP (r-TG). Toxic oligomers of h-IAPP form intracellularly in beta-cells of h-TG but not r-TG mice, and h-TG but not r-TG mice develop diabetes^25^. In the experiments presented in this study, we used mice in a pre-diabetic state to avoid the confounding effect of glucose toxicity (Supplemental Table 1). Increased expression of h-IAPP led to 81.2 ± 4% decrease in cytosolic p300, and 83.2 ± 11.2% decrease in nuclear protein levels of p300 in comparison to r-TG mice, as shown by subcellular fractionation (Fig. 2a and b). We conclude that p300 is downregulated in an animal model prone to develop diabetes, due at least in part to the propensity of h-IAPP to form toxic oligomers.

**Fig. 3:**
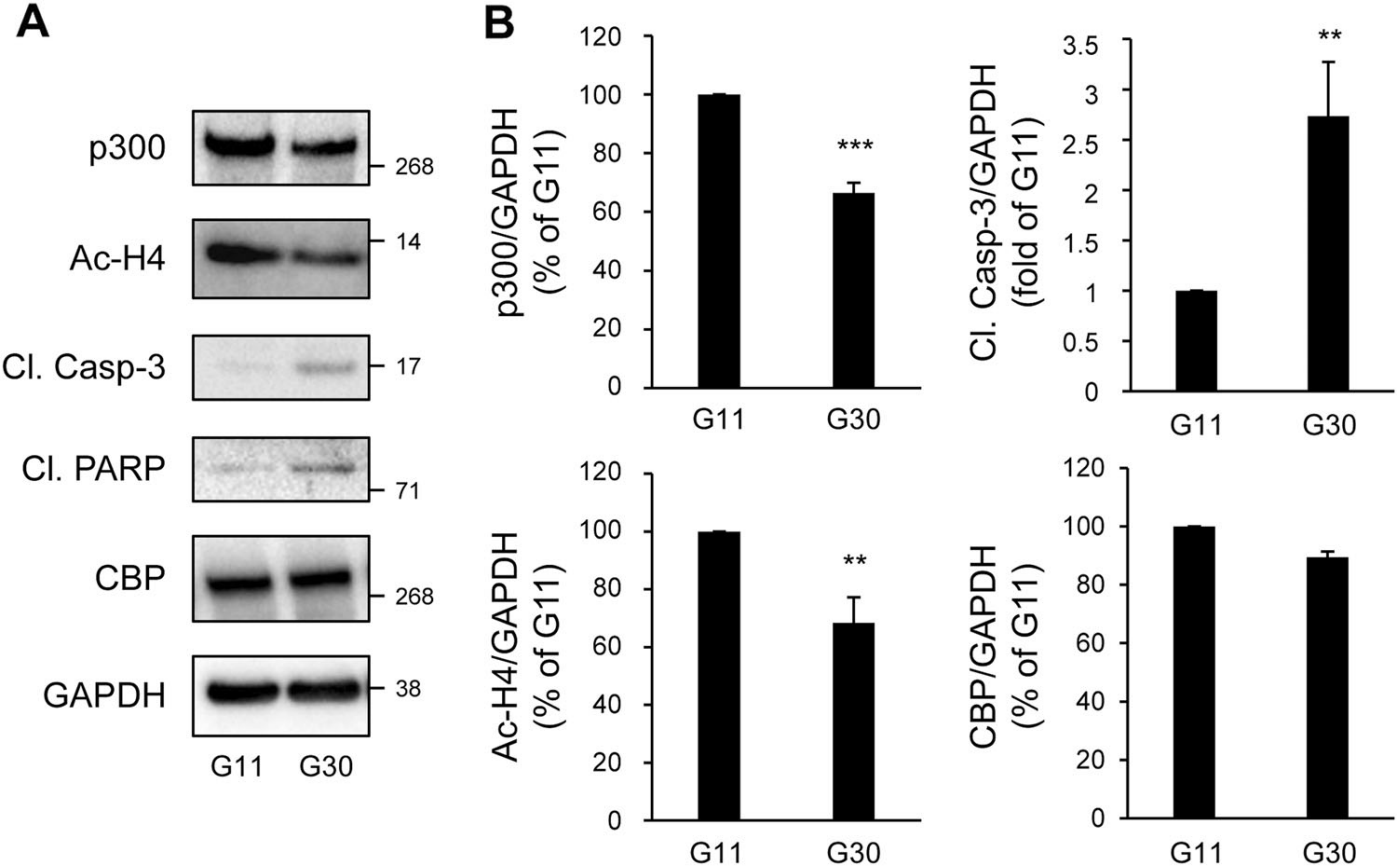
p300 and histone H4 acetylation levels are decreased under glucotoxicity in beta-cells. **a** INS-1E cells were exposed to 11 mM glucose (G11) or 30 mM glucose (G30) during 48 h. Protein levels of p300, acetyl-Histone H4 (Ac-H4), cleaved caspase-3 (Cl. Casp-3), cleaved PARP (Cl. PARP) and CBP (CREB-binding protein) were analyzed by western blot. GAPDH was used as loading control. **b** Quantification of p300, Ac-H4, Cl. Casp-3 and CBP protein levels. Data are expressed as mean ± SEM (*n* = 3); **P < 0.01, ***P < 0.001

**Fig. 4:**
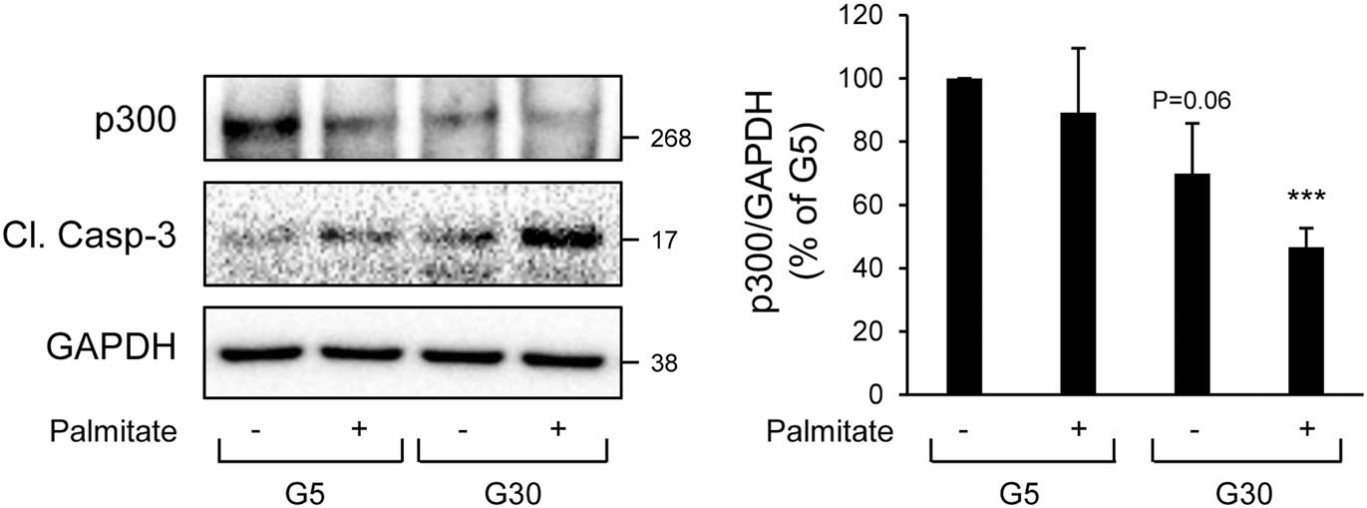
p300 protein levels are decreased under gluco/lipotoxicity in human islets. Human islets were exposed to 5 mM glucose + EtOH/BSA as vehicle (G5), 5 mM glucose + 0.5 mM palmitate, 30 mM glucose + EtOH/BSA as vehicle (G30) or 30 mM glucose + 0.5 mM palmitate during 72 h. Protein levels of p300 and cleaved caspase-3 (Cl. Casp-3) were analyzed by western blot. GAPDH was used as loading control. Data are expressed as mean ± SEM (*n* = 3); ***P < 0.001 vs G5

In T2D, chronic hyperglycemia is detrimental to beta-cells. Glucotoxicity led to increased beta-cell apoptosis, as shown by the cleavage of caspase-3 and PARP (poly(ADP-ribose) polymerase) (Fig. 3a and b). Treatment of INS-1E cells with 30 mM glucose for 48 h led to a 33.5 ± 3.4% decrease in p300 protein content (*P* < 0.001), while its paralog CBP (CREB binding protein) remained unaffected (Fig. 3a and b). Under these conditions, altered p300 protein levels were associated with decreased acetylation of p300’s targets Histones H4 (Fig. 3a and b), suggesting a decrease in p300 activity. To further confirm these results, isolated human islets were exposed for 72 h to high glucose. In human islets, p300 protein levels were decreased to the same extent as observed in INS-1E cells (30% decrease in p300 protein content; Fig. 3 and Fig. 4). Importantly, p300 protein level alteration was exacerbated under glucolipotoxicity conditions (30 mM glucose + 0.5 mM palmitate), as shown by the 53.3 ± 6% decrease in p300 protein levels (*P* < 0.001; Fig. 4) associated with the emergence of cleaved caspase-3 (Fig. 4).

**Fig. 5:**
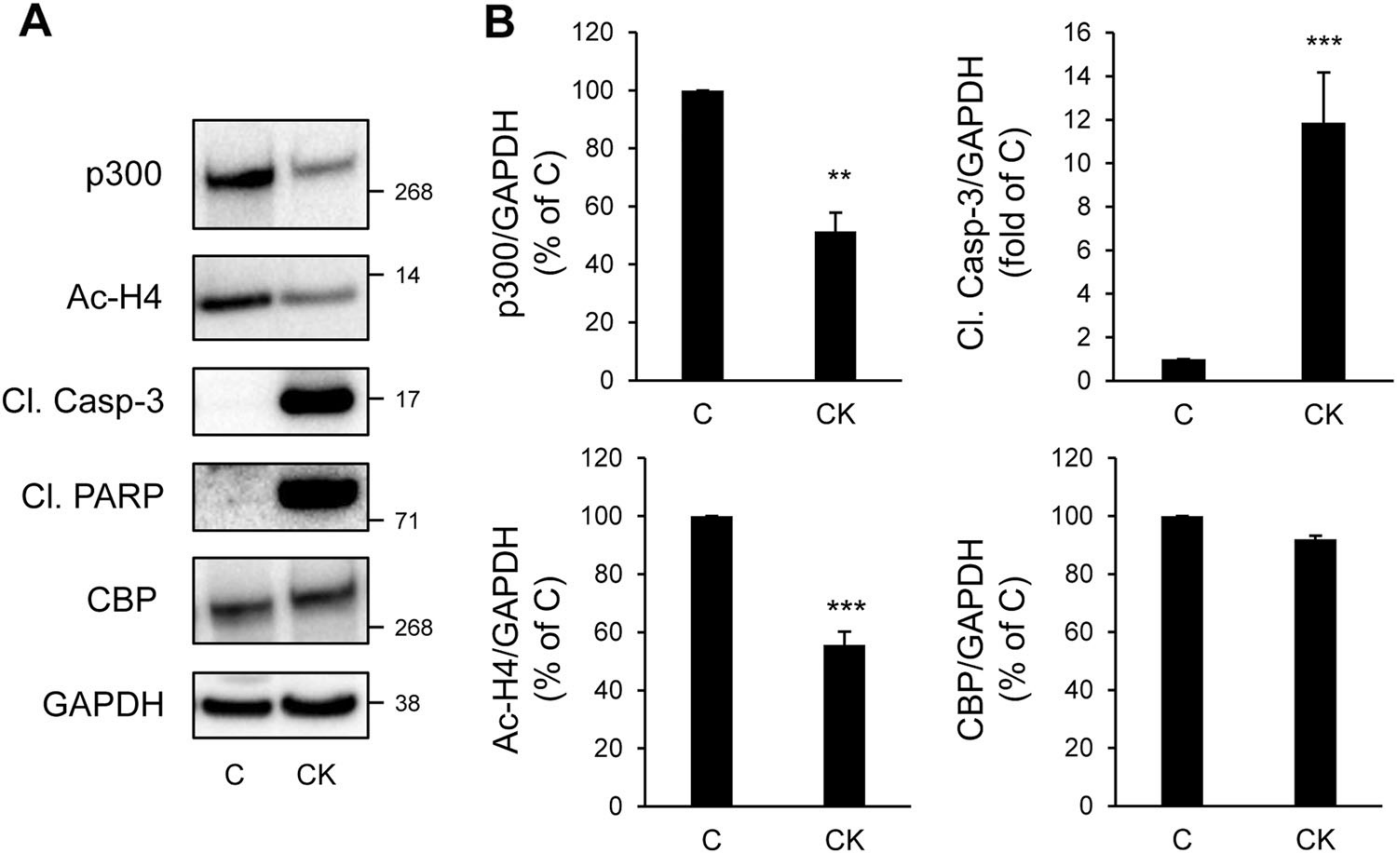
p300 and histone H4 acetylation levels are decreased under chronic exposure to pro-inflammatory cytokines in beta-cells. **a** INS-1E cells were exposed or not to pro-inflammatory cytokines mix (CK: 50 ng/ml TNFα, 0.2 ng/ml IL1β and 33 ng/ml IFNγfor 24 h (C, control). Protein levels of p300, acetyl-Histone H4 (Ac-H4), cleaved caspase-3 (Cl. Casp-3), cleaved PARP (Cl. PARP) and CBP were analyzed by western blot. GAPDH was used as loading control. **b** Quantification of p300, Ac-H4, Cl. Casp-3 and CBP protein levels. Data are expressed as mean ± SEM (*n* = 3); **P < 0.01, ***P < 0.001

Chronic inflammation is a hallmark of type 1 diabetes, and increased islet inflammation has also been reported in T2D, affecting both beta-cell mass and insulin secretion^26, 27^. Pro-inflammatory cytokines, particularly interleukin-1*β* (IL-1*β*), in combination with interferon-*γ* (IFN-*γ*) and/or tumor necrosis factor-*α* (TNF-*α*), lead to a decline in beta-cell function and survival. As expected, pro-inflammatory cytokines led to beta-cell apoptosis, as shown by the cleavage of caspase-3 and PARP (Fig. 5a and b). Treatment of INS-1E cells with the cytokine mixture for 24 h led to a 48.6 ± 6.5% decrease in p300 protein content, while its paralog CBP remained unaffected (Fig. 5a and b). As suggested by the decreased acetylation levels of Histones H4 (Fig. 5a and b), alteration in p300 protein levels was associated with a decreased in HAT activity.

Altogether, these data reveal that the diabetes environment alter p300 functional integrity in beta-cells.

### Diabetic conditions contribute to the proteasomal degradation of p300

**Fig. 6:**
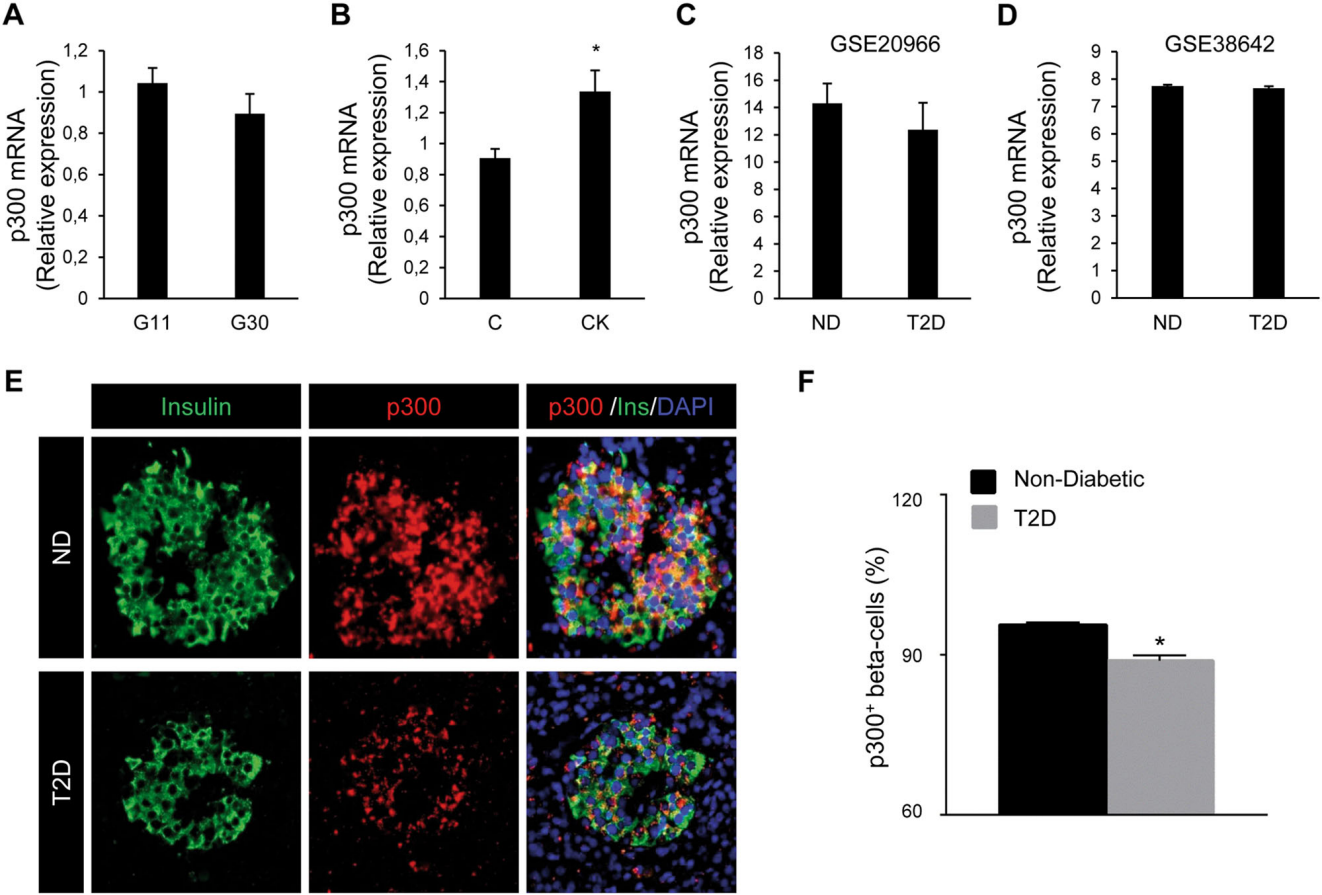
p300 mRNA levels are unchanged in INS-1E cells and human beta-cells/islets exposed to diabetic conditions but protein levels are decreased in beta-cells in T2D. Levels of p300 mRNA were determined by quantitative RT-PCR and normalized to the geometric mean of the expression levels of two housekeeping genes (*Tbp, rHprt*). **a** INS-1E cells cultured in 11 mM glucose (G11) or 30 mM glucose (G30) for 48 h. Data are expressed as mean ± SEM (*n* = 4). **b** INS-1E cells exposed or not to pro-inflammatory cytokines mix (CK: 50 ng/ml TNFα, 0.2 ng/ml IL1β and 33 ng/ml IFNγfor 24 h (C, control). Data are expressed as mean ± SEM (*n* = 3); *P < 0.05. **c**, **d** Beta-cell and islet transcriptomics analyses of p300 expression in T2D subjects compared to normal glycemic controls (ND) are based on two independent human data sets GEO: GSE20966 (ND; *n* = 10 and T2D; *n* = 10) and GEO: GSE38642 (ND; *n* = 55 and T2D; *n* = 9) respectively. Data are expressed as mean ± SEM. **e** p300 protein levels were assessed by immunofluorescence (p300, red; insulin, green; nuclei, blue) in human pancreatic tissue obtained at autopsy from non-diabetic subjects and subjects with type 2 diabetes. **f** Percentage of beta-cells positive for p300 in each group. ND Non-Diabetic, T2D Subjects with type 2 diabetes. Data are expressed as mean ± SEM; *P < 0.05

**Fig. 7:**
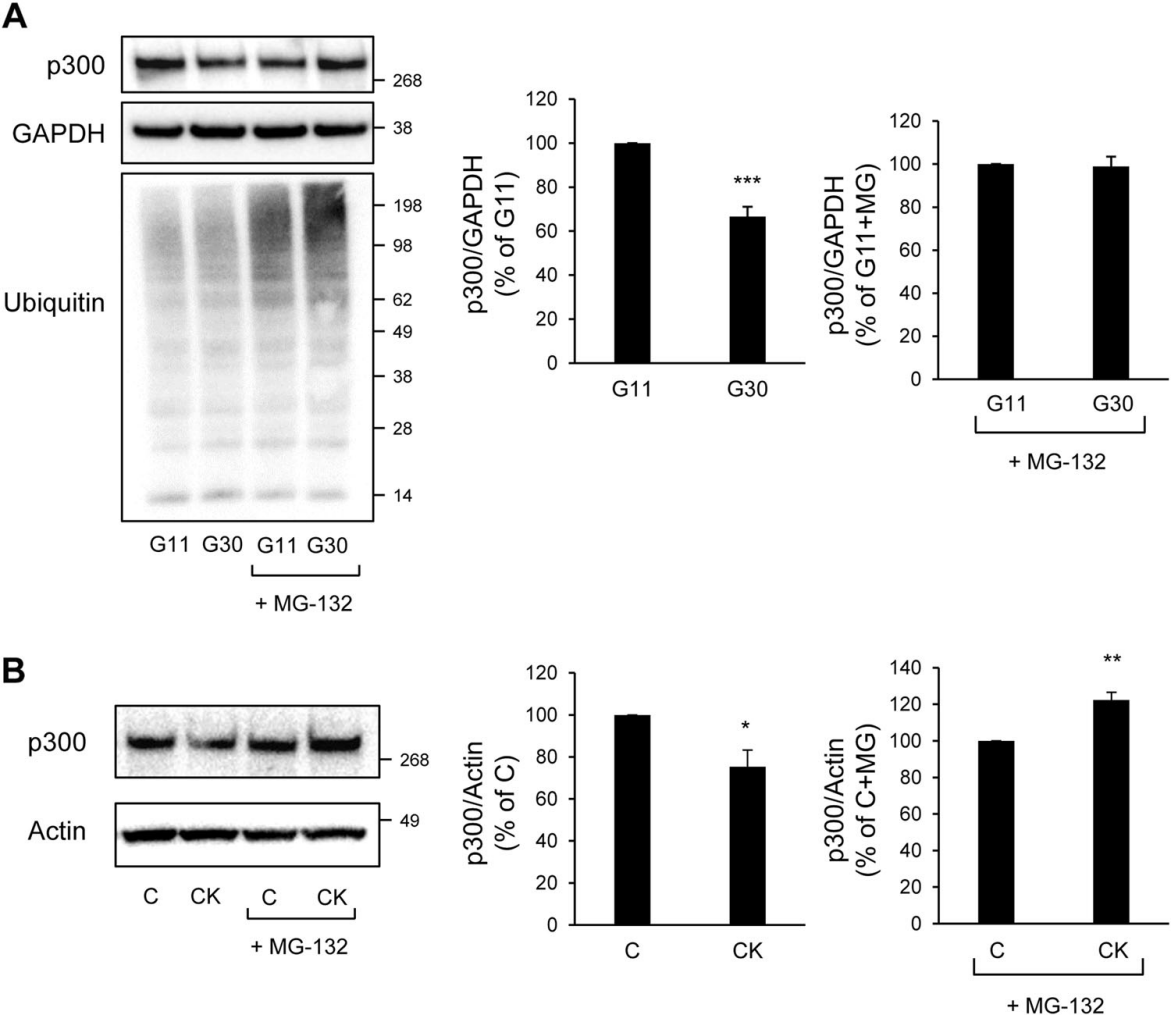
Glucotoxicity or pro-inflammatory cytokines-induced p300 loss is mediated by proteasomal degradation in beta-cells. **a** p300, Ubiquitin and GAPDH (loading control) protein levels in INS-1E cells cultured in 11 mM glucose (G11) or 30 mM glucose (G30) for 48 h in the presence or not of MG-132 (150 nM) for the final 8 h. The graphs represent the quantification of p300 protein levels (*n* = 3). **b** p300 and actin (loading control) protein levels in INS-1E cells exposed or not to pro-inflammatory cytokines mix for 24 h (C, control) in the presence of MG-132 (150 nM) for the final 8 h. The graphs represent the quantification of p300 protein levels. Data are expressed as mean±SEM (*n* = 3); *P < 0.05; **P < 0.01; ***P < 0.001

To delineate further the mechanisms involved in p300 protein loss under diabetogenic situations, we evaluated p300 gene expression. While p300 protein content was decreased (Figs. 3 and 5), we found that p300 mRNA levels were not altered in INS-1E cells exposed for 48 h to high glucose (Fig. 6a), and were even increased in INS-1E cells exposed for 24 h to pro-inflammatory cytokines (Fig. 6b). Interestingly, beta-cell and islet transcriptome analysis in T2D subjects from two independent data sets also revealed no change in p300 mRNA levels compared to normal glycemic controls (GEO: GSE20966^28^ and GEO: GSE38642;^29^ Fig. 6c and d, respectively). To evaluate whether p300 protein levels are altered in T2D, we examined pancreatic tissue from human subjects with T2D versus BMI-matched control subjects (Supplemental Table 2). The percentage of beta-cells positive for p300 was decreased in subjects with T2D (*P* < 0.05; Fig. 6e and f). These data therefore suggest that, under diabetes-associated conditions, the decrease in p300 protein expression in beta-cells occurs at a post-transcriptional level. Many transcriptional factors and activators are regulated by the 26S proteasome, which is one of the major proteolysis systems of the cell and localizes to both the cytoplasmic and nuclear compartments. Among other, proteasomal degradation has been reported as a mechanism involved in p300 turnover^30^. We evaluated the levels of p300 content in INS-1E cells exposed to high glucose for 48 h and treated for the last 8 h with or without the proteasome inhibitor MG-132 (150 nM). As expected, treatment with MG-132 led to accumulation of ubiquitinated proteins in treated cells (darken smears, Fig. 7a), confirming proteasome inhibition. This treatment totally prevented p300 protein decrease induced by high-glucose exposure (Fig. 7a), indicating that glucotoxicity induces a proteasomal degradation of p300. Similarly, treatment of cells with MG-132 prevented p300 protein loss induced by the pro-inflammatory cytokines (Fig. 7b), showing that the mechanism subserving p300 alteration upon cytokine exposure is also a proteasome-dependent degradation. Altogether the data obtained with INS-1E cells and human beta-cells/islets point to a proteasomal degradation involved in p300 loss in pathological beta-cells.

### Activation of melatonin signaling restores p300 levels in beta-cells exposed to diabetic situations

**Fig. 8:**
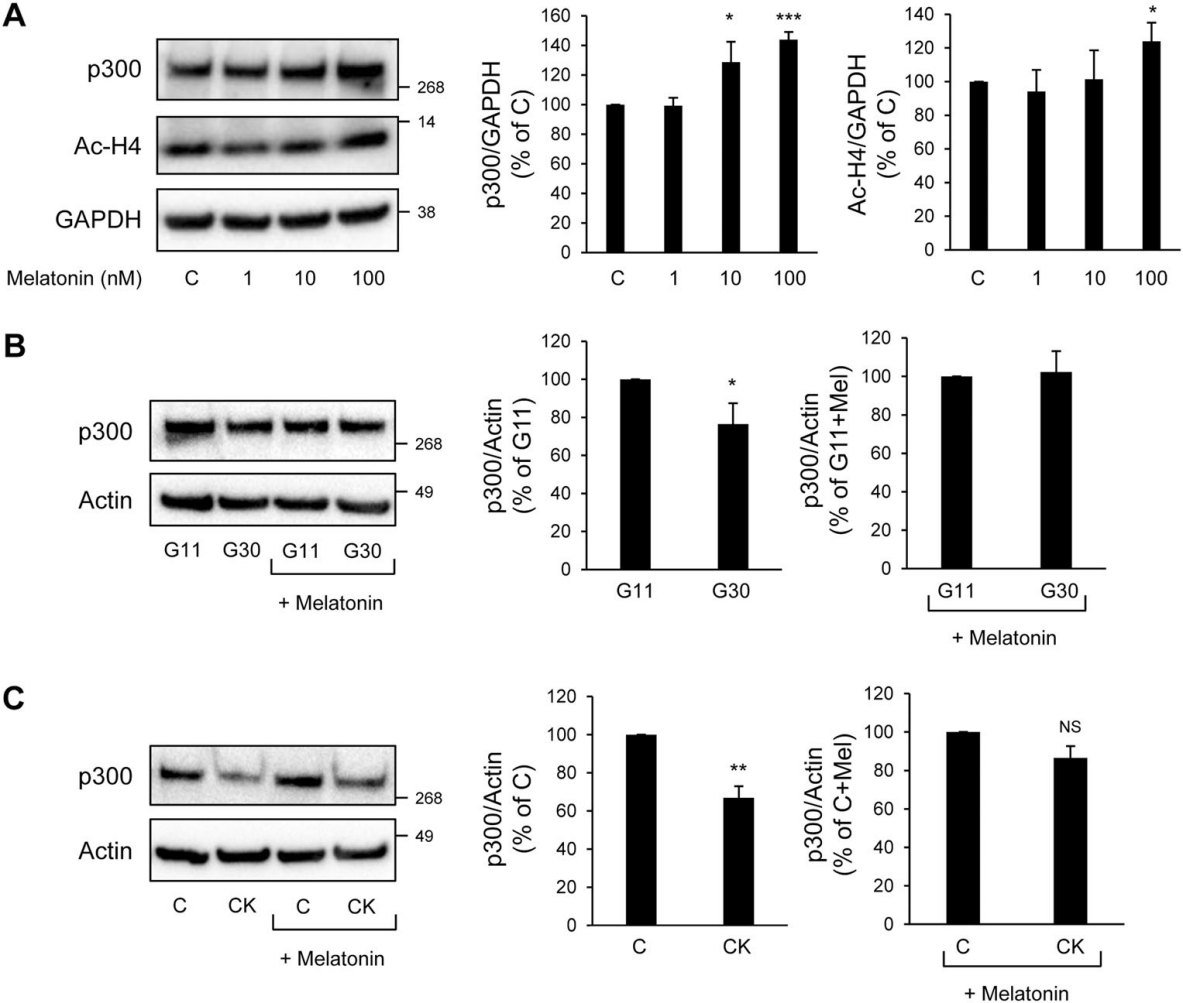
Activation of melatonin receptor signaling preserves p300 levels in beta-cells exposed to diabetes-related stress. **a** INS-1E cells were exposed to 1, 10, 100 nM melatonin during 24 h (C, control cells). Protein levels of p300 and acetylated histone H4 (Ac-H4) were analyzed by western blot. GAPDH was used as loading control. The graphs represent the quantification of p300 and Ac-H4 protein levels (*n* = 3–4). **b** p300 and actin (loading control) protein levels in INS-1E cells cultured in 11 mM glucose (G11) or 30 mM glucose (G30) and exposed for the final 14 h to media containing melatonin (100 nM) or not. The graphs represent the quantification of p300 protein levels (*n* = 3). **c** p300 and actin (loading control) protein levels in INS-1E cells exposed or not to pro-inflammatory cytokines mix (CK: 50 ng/ml TNFα, 0.2 ng/ml IL1β and 33 ng/ml IFNγ for 24 h (C, control) and incubated for the final 14 h to media containing melatonin (100 nM) or not. The graphs represent the quantification of p300 protein levels (*n* = 3). Data are expressed as mean ± SEM; *P < 0.05, **P < 0.01, ***P < 0.001. NS, non-significant

Melatonin has been recently identified as a beta-cell protective hormone^31–34^. Interestingly, melatonin’s actions are purported to be mediated through connection with the proteasomal degradation^35, 36^ and an increase in p300 expression^37^ as demonstrated in other cell types. We therefore investigated the possible effect of melatonin on p300 levels in beta-cells. Exposure of INS-1E cells to melatonin (in concentration ranges 1–100 nM for 24 h) led to increased p300 protein levels associated with increased acetylation levels of Histones H4, particularly evident at 100 nM melatonin concentration (Fig. 8a). We therefore used a 100 nM melatonin concentration to investigate whether melatonin would restore p300 levels in beta-cells exposed to diabetic conditions. We evaluated p300 protein levels in INS-1E cells exposed to either high glucose for 48 h or the cytokines mixture for 24 h, and incubated with melatonin for the final 14 h of culture. We found that melatonin exposure preserved p300 protein levels under high glucose conditions (Fig. 8b), and was also protective against its loss induced by the pro-inflammatory cytokines (Fig. 8c). Given the newly discovered role of p300 in beta-cell survival and the well-described role of melatonin in beta-cell protection under diabetic conditions^33^, our data point to p300 as a new link between melatonin signaling and beta-cell protection in T2D.

## Discussion

Our study reveals for the first time that the histone acetyl transferase p300 plays a key role in beta-cell survival as demonstrated by the emergence of apoptosis upon knockout or inhibition of p300. Both p300 enzyme acetylation activity and protein binding activity therefore seem important for beta-cell protection. In addition, data obtained from the INS-1E beta-cell line as well as mouse and human islets show that diabetes-related cytotoxic conditions (proteotoxicity, glucotoxicity, lipotoxicity, and inflammation) adversely affect p300 protein levels and function. Our results unravel a new mechanism for glucotoxicity- and cytokines-induced beta-cell apoptosis involving proteasomal degradation of p300. Finally, we found that activation of melatonin signaling preserves p300 levels upon glucotoxicity and inflammation.

Among the mechanisms involved in p300 regulation, proteasome-dependent degradation has been well described in several studies and cell types^30, 38, 39^. The data obtained using the INS-1E beta-cell line revealed the involvement of this degradative pathway to downregulate p300 under pathological conditions. Importantly, the decreased p300 protein levels in beta-cells of human subjects with T2D, confronted with the data obtained from human data sets showing similar p300 mRNA levels in beta-cells/islets from T2D and non-diabetic subjects, further supports a post-transcriptional regulation and degradation of p300. Regarding the mechanism targeting p300 to degradation, it has been shown that phosphorylation of p300 by the pro-apoptotic kinase p38-MAPK is a mechanism by which p300 undergoes proteasomal degradation^38, 39^. Glucotoxicity and exposure to pro-inflammatory cytokines, known to promote p38-MAPK activation in beta-cells^33, 40^, may therefore lead to p300 phosphorylation and subsequent degradation.

Melatonin is a hormone produced and secreted from the endocrine cells in the pineal gland and exhibits nocturnal production and secretion pattern. Key roles of melatonin on beta-cell health and glucose homeostasis are now confirmed by several recent studies^31–34^. The pathogenesis of T2D is also associated with impaired melatonin production and secretion^34, 41^. In addition, genome-wide association scan studies have reported that a variance in the gene encoding melatonin receptor 2 (*MTNR1B*) is associated with an increased risk of beta-cell failure and T2D^42, 43^. Interestingly, melatonin has been shown to decrease glucotoxicity-induced activation of p38-MAPK in beta-cells to promote beta-cell survival^33^. One can thus speculate that glucotoxicity (or cytokine exposure)-induced p38-MAPK activation would lead to p300 phosphorylation to promote its proteasomal degradation, a mechanism likely to be blocked by melatonin to protect beta-cells.

Similar to the role of p300 in neuron survival^44, 45^, our study demonstrates a key role of p300 in beta-cell survival. Among the genes involved in beta-cell survival, recent studies suggest that the circadian clock is essential for beta-cell functional integrity^46–49^. Since p300 has been described to modulate clock genes BMAL1/CLOCK transactivation ability^10^, we can consider that p300 maintains beta-cell survival via a positive modulation of the circadian clock. Moreover, since melatonin also regulates clock gene expression in beta-cells^50, 51^, we can hypothesize that melatonin signaling would favor beta-cell clock gene expression and activation, at least in part, through stabilization of p300 to ultimately protect beta-cells from cytotoxic injury.

Whereas p300 plays an important role in beta-cell survival and seems therefore controlling expression of genes crucial for such purpose, we cannot exclude that specific environments and interacting partners will target p300 to other genes in beta-cells. Indeed, it has been reported that glucose stimulates the recruitment of p300 to the promoter region of the Txnip gene in human islets^52^ and that p300 knockout prevents the expression of this pro-apoptotic factor Txnip in beta-cells exposed to high glucose^53^. Whereas the study from Bompada et al.^53^ aimed to investigate the role of p300 knock-out under pathological conditions (i.e., glucotoxicity), we rather questioned the role of p300 knock-down or inhibition under physiological conditions. This may therefore explain the discrepancy between our results and the above-mentioned studies^52, 53^. Nevertheless, consistent with the role of p300 in Txnip gene expression in beta-cells^52, 53^, evaluation of Txnip expression under p300 knock-down or inhibition revealed that the basal levels of Txnip were diminished (Supplemental Fig. 5). Despite this decrease in the pro-apoptotic factor Txnip, we clearly detected apoptosis under these conditions. Thus, it is likely that knock-down/inhibition of p300 under normal conditions blocks not only the expression of pro-apoptotic factors such as Txnip, but also the expression of survival factors which overall favors the emergence of beta-cell apoptosis. On the other hand, since our glucotoxicity conditions led to a partial decrease in p300, the remaining pool of p300 available may thus be redirected to control other genes such as Txnip. At last, our study and the one from Bompada et al.^53^. agree that blockade of p300 under normal condition leads to beta-cell apoptosis and altered insulin response to glucose (decreased stimulation index with elevated basal insulin secretion). Basal insulin secretion and the subsequent inability to further increase insulin secretion in response to glucose is not uncommon in individuals with T2D. Further investigations are required to determine the potential involvement of p300 in insulin gene expression and/or insulin exocytosis.

Although p300 and CBP are highly homologous proteins (63% homology at the amino acid level) and have some interaction partners in common, they have distinct functions and cannot always replace each other^9^. Interestingly, our results show that glucotoxicity or cytokines specifically reduced p300 protein levels, without inducing any decrease or increase in CBP to compensate p300 loss, suggesting specificity in the action of high glucose and cytokines to alter p300 in beta-cells. While the literature often fails to distinguish between p300 and CBP (or other HATs), additional studies are required to identify genes that are specifically controlled by p300 and to determine p300-interacting partners in beta-cells in vivo.

Loss-of-function mutations in *EP300* (p300) or *CREBBP* (CBP) are known causes of the Rubistein-Taybi syndrome, a rare congenital developmental disorder^54^. As mentioned in earlier articles, few patients with Rubistein-Taybi syndrome developed early onset glucose phenotypes^55, 56^. It would therefore be of great interest to follow glucose regulation in a larger cohort of Rubistein-Taybi syndrome patients with specific p300 mutations to further ascertain association between p300 loss and diabetes-like phenotypes in humans.

Our study demonstrates for the first time a key role of p300 in beta-cell survival and function and its alteration under pathological situations. We further show that p300 proteasomal degradation plays a role in the pathophysiology of diabetes and constitutes a potential site for therapeutic intervention. Finally, melatonin signaling may represent a strategy for the maintenance of p300 integrity in order to preserve a functional beta-cell mass in T2D.

## Materials and methods

### Animal models

C57BL/6J mice were purchased from Charles River (L’Arbresle, France). All experiments were performed using 4-month-old male mice, except when indicated. All animal studies complied with the animal welfare guidelines of the European Community and were approved by the Direction of Veterinary Departments of Hérault and Nord, France (59-350134).

Transgenic mice were bred and housed at the University of California, Los Angeles (UCLA) animal housing facility. The institutional animal care and use committee of the UCLA approved all experimental procedures. Animals were maintained on a 12-h day/night cycle with Harlan Teklad Rodent Diet 8604 (Madison, WI, USA) and water ad libitum. Males were used for the experiments. The generation and characterization of transgenic mice homozygous for human-IAPP (h-TG: FVB-*Tg(IAPP)6Jdm/Tg(IAPP)6Jdm*) and rodent-IAPP (r-TG: FVB/N-*Tg(Iapp)6Wcs/Tg(Iapp)6Wcs*) have been described previously^57^. Control WT (FVB) mice were originally purchased from Charles Rivers Laboratory (Wilmington, MA, USA) and bred at UCLA. Characteristics of mice used for the experiments are listed on Supplemental Table 1.

### Mouse islet isolation

Islets were isolated from mice after collagenase digestion of the pancreas^58^, and were used either immediately or after overnight culture. Islets were washed with ice-cold PBS and lysed in NP40 lysis buffer (0.5% Nonidet P-40, 20 mM Tris-HCl, pH 7.5, 150 mM NaCl, 2 mM MgCl2, 1 mM dithiothreitol, 5 mM NaF, 1 mM Na_3_VO_4_, and protease inhibitors [Sigma-Aldrich, St. Louis, MO, USA]). After 10 min of incubation in lysis buffer on ice, islets were sonicated for 10 s and centrifuged at 10,000 r.p.m. at 4 °C for 10 min to remove insoluble materials. Supernatant was stored at −20 °C until use for subsequent protein determination by BCA assay (Bio-Rad, Marnes-la-Coquette, France) and western blotting.

In the experiments testing the effect of p300 inhibitor, islets were used after an overnight culture in RPMI-1640 medium containing 11 mM glucose supplemented with 10% heat-inactivated FBS, 2 mM glutamine, 10 mM HEPES, 100 IU/ml penicillin and 100 μg/ml streptomycin (Life technologies, Courtaboeuf, France). Islets were then treated with 30 μM C646 (Merck, Fontenay-sous-Bois, France) for 48 h. To minimize the effects of subjective bias, groups of islets were randomly distributed in tubes. No blinding was done. Immunostaining in islets is described in Supplemental materials and methods.

### Human islets

Experiments involving usage of human islets were performed in agreement with the local ethic committee (CHU, Montpellier) and the institutional ethical committee of the French Agence de la Biomédecine (DC Nos. 2014-2473 and 2016-2716). Informed consent was obtained from all donors. Pancreases were harvested from three brain-dead non-diabetic donors. Isolated islets were prepared by collagenase digestion followed by density gradient purification at the Laboratory of Cell Therapy for Diabetes (Institute for Regenerative Medicine and Biotherapy, Montpellier, France), according to a slightly modified version of the automated method^59^. Following isolation, human islets were cultured for recovery for 3 days at 37 °C, in a 5% CO_2_ atmosphere, in CMRL 1066 medium (Life Technologies) containing 5.6 mM glucose supplemented with 10% FBS, 2 mM glutamine, 100 IU/ml penicillin, and 100 μg/ml streptomycin. Islets were then incubated for 72 h in CMRL 1066 medium (without FBS) containing 5.6 mM or 30 mM glucose ± 0.5 mM palmitate (see palmitate preparation in Mancini et al.^60^). At the end of the experiment, islets were washed with cold PBS and lysed for 10 min at 4 °C in NP40 lysis buffer, sonicated for 10 s and centrifuged at 10,000 r.p.m. for 10 min.

### Human pancreatic sections and immunostaining

Human pancreas was procured from the Mayo Clinic autopsy archives with approval from the Institutional Research Biosafety Board. Informed consent was obtained from all donors. Clinical characteristics of human donors are listed on Supplemental Table 2. Paraffin-embedded pancreatic sections were co-immunostained by immunofluorescence for insulin (ab7842; 1:100; Abcam, Cambridge, MA, USA), p300 (sc-48343; 1:100; Santa Cruz Biotechnology, Dallas, TX, USA) and cover slipped with Vectashield-DAPI mounting medium (Vector Laboratories, Burlingame, CA, USA), stored in dark at 4 °C, and analyzed within 1–3 days after staining. Blinded slides were viewed, imaged and analyzed using a Zeiss Axio Observer Z1 microscope (Carl Zeiss Microscopy LLC, NY, USA) and ZenPro software (Carl Zeiss Microscopy, LLC). To quantify percentage of p300 positive beta-cells, 500 beta-cells per pancreatic section were examined in detail and counted at ×20 magnification for the presence/absence of p300 immunoreactivity.

### Cell culture

The rat beta-cell line INS-1E was provided by Dr. P. Maechler (Department of Cell Physiology and Metabolism, University of Geneva, Geneva, Switzerland)^61^. INS-1E cells were grown in RPMI-1640 medium with 11 mM glucose supplemented with 7.5% heat-inactivated FBS, 1 mM sodium pyruvate, 50 μM *β*-mercaptoethanol, 2 mM glutamine, 10 mM HEPES and 100 IU/ml penicillin and 100 μg/ml streptomycin (Life Technologies) at 37 °C in a humidified 5% CO_2_ atmosphere. No mycoplasma contamination was detected.

-For glucotoxicity experiments, INS-1E cells were cultured in complete RPMI 1640 medium (Life Technologies) containing 11 or 30 mM glucose for 72 h.
-For pro-inflammatory cytokine exposure, INS-1E cells were incubated in a cytokine mix (100 IU/ml IL-1*β* (0. 2 ng/ml), 500 IU/ml TNF-*α* (50 ng/ml) and 100 IU/ml IFN-*γ* (33 ng/ml) for 24 h. Murine recombinant IFN-γ were from Invitrogen (Life Technologies), murine IL-1*β* and TNF-*α* from PeproTech.

The proteasome inhibitor MG-132 (dissolved in DMSO; Millipore, Saint-Quentin-en-Yvelines, France) was added at 150 nM during the last 8 h of the treatment. Melatonin 100 nM (dissolved in DMSO; Bachem, Weil AM Rhein, Allemagne) was added during the last 14 h of the treatment.

-In the experiments testing the effect of p300 inhibitor, cells were treated with 30 μM C646 (dissolved in DMSO) for 24 h.

At the end of the experiment, cells were washed with cold PBS and lysed for 10 min at 4 °C in NP40 lysis buffer and centrifuged at 10,000 r.p.m. for 10 min.

### p300 siRNA

p300 expression was silenced in INS-1E cells using Silencer Select siRNA duplexes designed for rat *Ep300* (s220367, Life Technologies). Cells were seeded in 6-well plates at 800,000 cells/well and grown overnight to reach 40–50% confluency. The next day, lipofectAMINE3000-siRNA complexes were prepared according to the manufacturer’s instructions. p300 siRNA duplexes were tested at final concentrations of 10, 25 or 50 nM. Cells were transfected with p300 siRNA or control siRNA (scramble) in Opti-MEM (Life Technologies) for 24 h before switching to fresh culture medium. After 48 or 72 h of transfection, cells were washed with cold PBS and lysed for 10 min at 4 °C in NP40 lysis buffer and centrifuged at 10,000 r.p.m. for 10 min.

### Western blotting

Proteins (25–50 μg/lane) were separated on a 4–12% Bis-Tris (or 3-8% Tris-Acetate) NuPAGE gel and blotted onto a PVDF membrane (FluoroTrans; VWR, Fontenay-sous-Bois, France). Membranes were probed overnight at 4 °C with primary antibodies against cleaved caspase-3 (Cell signaling, Leiden, Netherlands, 9661), cleaved PARP (Cell signaling, 9542), CBP (Cell signaling, 7389), Ubiquitin (Cell signaling, 3936), Phospho-Histone H2A.X (Ser 139; Cell signaling, 9718), phosho-CREB (Ser 133; Cell signaling, 9198) and GAPDH (Cell signaling, 5174), p300 (Millipore, 05-257), acetyl-Histone H3 (Millipore, 06-599), acetyl-Histone H4 (Millipore, 06-866), Actin (Sigma, A5441), Txnip (Cell signaling, 14715), Pdx1 (Cell signaling, 5679), Nkx6.1 (Cell signaling, 54551). Horseradish peroxidase-conjugated secondary antibodies were from Cell signaling. Proteins were visualized by enhanced chemiluminescence (Millipore) on ChemiDoc camera (Bio-Rad) and protein expression levels were quantified using the ImageJ software.

### RNA isolation, RT-PCR, real-time quantitative PCR

Total RNA was extracted using the RNeasy Mini Kit (Qiagen, Courtaboeuf, France) performing on-column DNase digestion with RNase-Free DNase Set (Qiagen) according to the manufacturer’s instructions. RNA (1 μg) was used for preparation of single-stranded cDNA using Superscript III Reverse transcriptase (Life Technologies) by the oligo-dT priming method. Real-time quantitative PCR was performed with the LightCycler FastStart DNA Master SYBR Green I kit (Roche, Meylan, France) and the LightCycler PCR equipment (Roche). The oligonucleotide primers were: 5′-GAACAAGGGCATTTTGCCA-3′ + 5′-TAGCGAGCTGTGAAAGCATTGA-3′ for rat *Ep300*. All measurements were normalized to the geometric mean of the expression levels of two housekeeping genes: rat *Hprt* (5′-TGACTATAATGAGCACTTCAGGGATT-3′ + 5′-TCGCTGATGACACAAACATGATT-3′) and rat *Tbp* (5′-GTTGACCCACCAGCAGTTCAG-3′ + 5′-AATCCAGGAAATAATTCTGGCTCATA).

### Insulin secretion

Cells were pre-incubated for 2 h in KRB buffer^58^ containing 1.4 mM glucose, followed by a 1h incubation at 1.4 mM or 16.7 mM glucose. Supernatant from the incubation buffers were collected and cleared by centrifugation. Insulin content extraction was performed using acid ethanol. Insulin release and contents were measured by Homogenous Time Resolved Fluorescence (HTRF) (Cisbio bioassays, Codolet, France) according to the manufacturer’s instructions. HTRF signals were measured using Pherastar FS (BMG Labtech, Ortenberg, Germany) microplate reader. Insulin release was then normalized to insulin content. The insulin stimulation index was calculated as the ratio of stimulated to basal insulin secretion, both of which are normalized to insulin content

### Human islet gene expression

To identify transcriptomic datasets from human pancreatic islets, GEO analysis from the NCBI was performed using “human islet T2D” as keywords and filtered with “Datasets”. Two datasets were selected based on the highest number of samples (GEO: GSE38642;^29^ and GEO: GSE20966^28^). Datasets were downloaded from the GEO and analyzed.

### Statistical analysis

Results are expressed as the means±SEM. for *n* independent experiments, as indicated in figure legends. Statistical analyses were carried out using Student’s *t*-test or one-way ANOVA followed by Sidak’s post hoc test for multiple comparisons using GraphPad Prism 7. A *P-*value of < 0.05 was taken as evidence of statistical significance (^*^P < 0.05, ^**^P < 0.01, ^***^P < 0.001).

## Electronic supplementary material

Supplemental Tables and Figures

Supplemental Materials and Methods
