# Supplementary material for: Proteasomal degradation of the histone acetyl transferase p300 contributes to beta-cell injury in a diabetes environment
Source: Cell Death Dis. 2018 May 22;9(6):600. doi: 10.1038/s41419-018-0603-0 (PMC5964068; doi:10.1038/s41419-018-0603-0)
Supplement: Supplementary file 1 — Supplemental Tables and Figures [file 41419_2018_603_MOESM1_ESM.pdf]

| Mice | n | Age (wks) | Weight (g) | Fasting glucose (mg/dl) |
|---|---|---|---|---|
| **WT** | 6 | 9-10 | 24.2 ± 0.4 | 76.6 ± 6.5 |
| **r-TG** | 6 | 9-10 | 24 ± 0.4 | 44.5 ± 4.1 |
| **h-TG** | 4 | 9-10 | 23.2 ± 0.5 | 79.2 ± 7.6 |

**Supplemental Table 1.** Characteristics of mice used for islet subcellular fractionation in Fig.2.

| Human donors | n | Age (years) | BMI ($kg/m^2$) | Fasting plasma glucose (mg/dl) | HbA1c (%) |
|---|---|---|---|---|---|
| **Non-Diabetic** | 4 | 75 ± 6 | 25 ± 1 | 82 ± 4 | n/a |
| **Type 2 Diabetes** | 4 | 78 ± 6 | 33 ± 1 | 196 ± 31 | 7 ± 1 |

**Supplemental Table 2.** Clinical characteristics of human donors used for immunostaining analysis of p300 in Fig.6. Values are mean ± SE.

**A Mouse islets** **B**

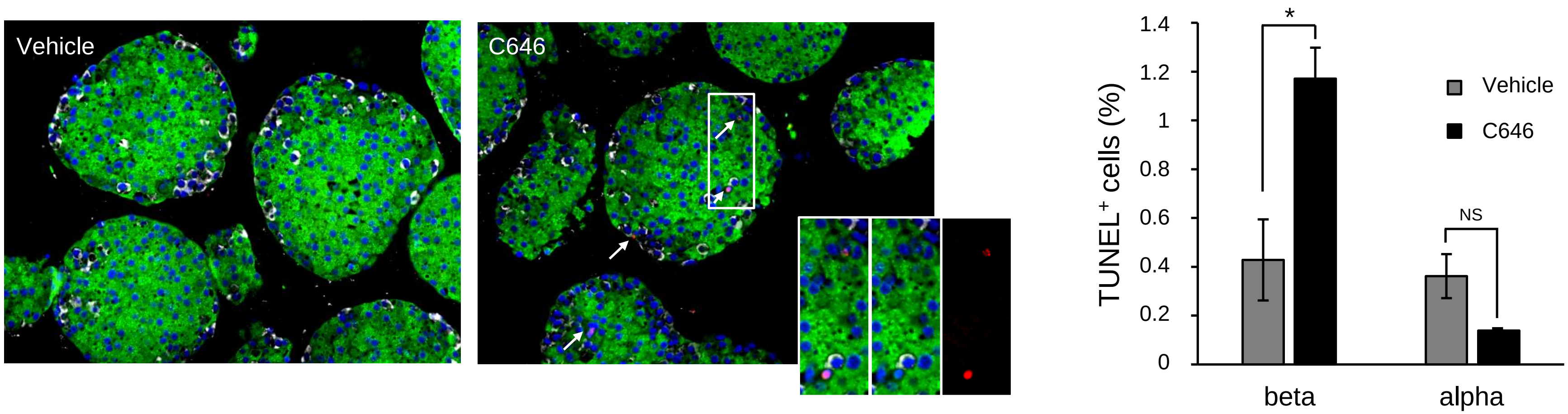


**C Human islets**

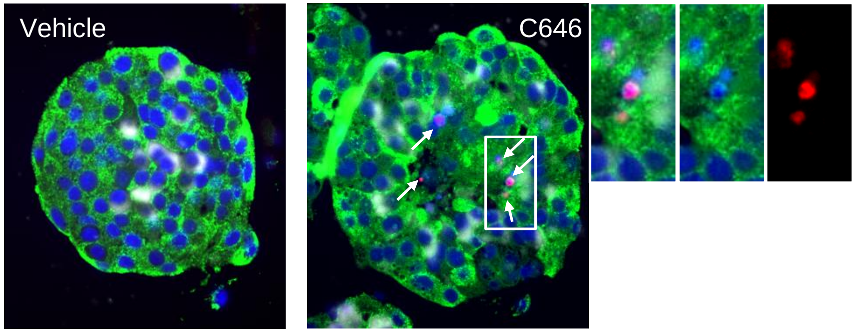


**D**

| Human donors | TUNEL⁺ beta-cells | | | TUNEL⁺ alpha-cells | | |
|---|---|---|---|---|---|---|
| | Vehicle | C646 | **fold** | Vehicle | C646 | **fold** |
| **HI-1** | 2.2 % | 3.8 % | **1.7** | 0.39 % | 0.41 % | **1.1** |
| **HI-2** | 0.27 % | 0.4 % | **1.5** | 0.19 % | 0.17 % | **0.9** |

**Supplemental Figure 1. Inhibition of p300 leads to beta-cell apoptosis in mice and humans. (A)** TUNEL staining was assessed by immunofluorescence (TUNEL, red; insulin, green; glucagon, white; nuclei, blue) in isolated mouse islets treated or not with C646 (30 μM for 72h). **(B)** Percentage of beta-cells and alpha-cells positive for TUNEL in each group. Data are expressed as mean ± SEM; *P < 0.05. **(C)** TUNEL staining was assessed by immunofluorescence (TUNEL, red; insulin, green; glucagon, white; nuclei, blue) in human islets treated or not with C646 (30 μM for 72h) (n=2 human donors). **(D)** Percentage of beta-cells and alpha-cells positive for TUNEL for each human donor (HI-1 and HI-2) in each group. The fold of change indicated is *versus* “Vehicle”.

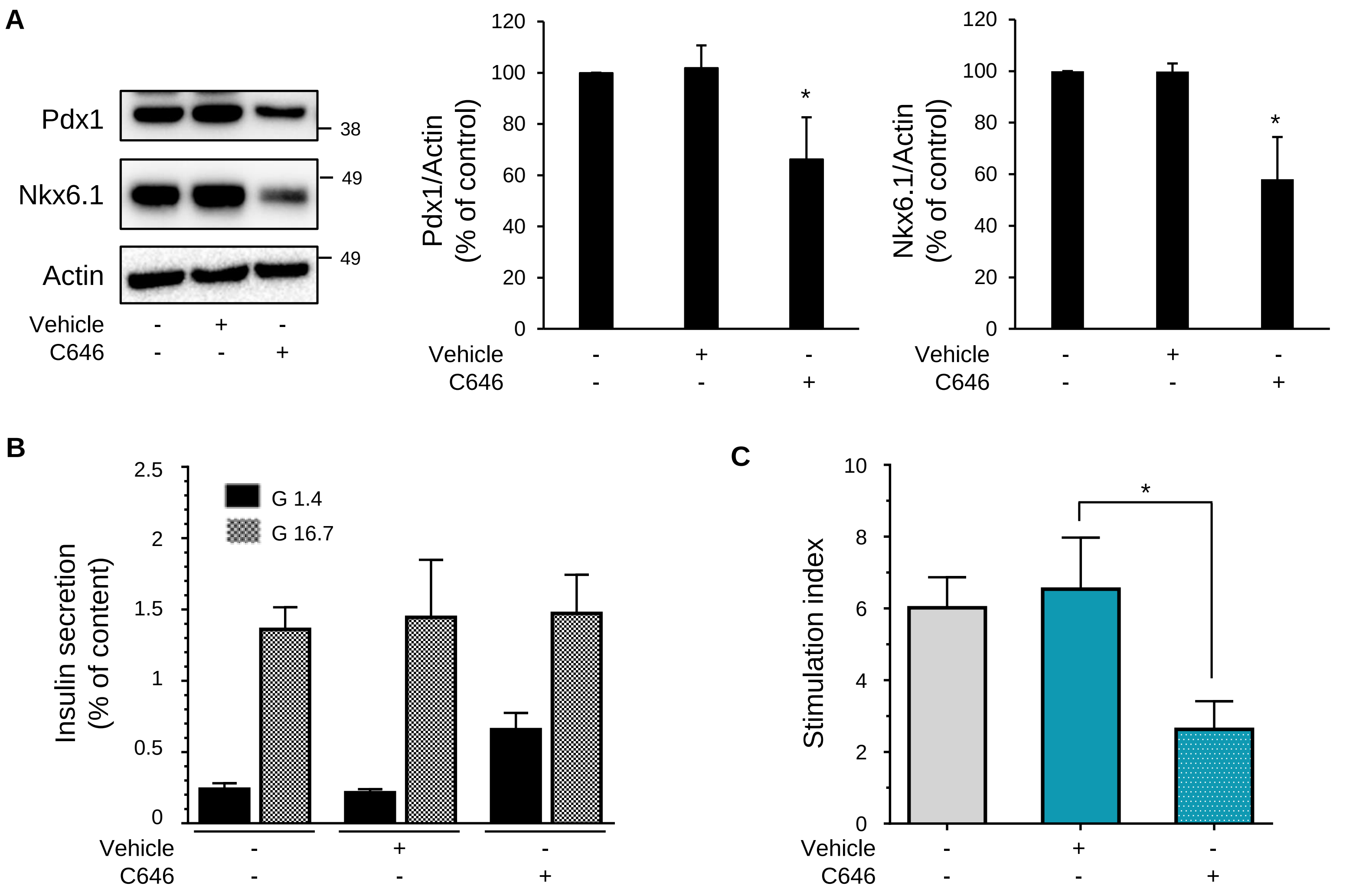


**Supplemental Figure 2. Inhibition of p300 by C646 alters beta-cell function. (A)** INS-1E cells were treated with C646 (30 µM for 24h) (or with 0.003% DMSO as vehicle). Levels of Pdx1 and Nkx6.1 were assessed by western blot. Actin was used as loading control. The graphs represent the quantification of the western blots (n=3). Data are expressed as mean ± SEM; *P<0.05 *vs* Vehicle. **(B)** INS-1E cells were treated with C646 (30 µM for 24h) (or with 0.003% DMSO as vehicle). Following a 2-h quiescent period in Krebs 1.4 mM glucose (G1.4), cells were stimulated with 16.7 mM glucose (G16.7) during 1h at 37°C; (G1.4 refers to non-stimulated cells). Insulin secretion and insulin content were measured by HTRF (Homogeneous Time Resolved Fluorescence). The insulin content remained unchanged. The graph represents insulin secretion normalized to insulin content. **(C)** The graph represents the stimulation index (ratio G16.7: G1.4). Data are expressed as mean ± SEM (n=4); *P<0.05.

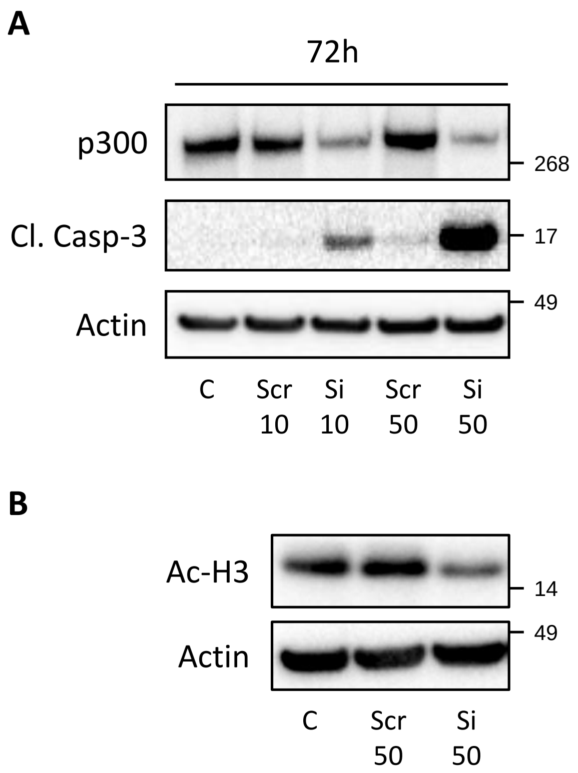


**Supplemental Figure 3. Knock-down of p300 by siRNA (72h) leads to beta-cell apoptosis. (A)** INS-1E cells were transfected with scramble (Scr) or p300 siRNA (Si) (10 or 50 nmol/L as indicated) during 72h; (C, non-transfected cells). p300 and cleaved caspase-3 (Cl. Casp-3) protein levels were analyzed by western blot. Actin was used as loading control. **(B)** Acetyl-Histone H3 (Ac-H3) protein levels were analyzed by western blot. Actin was used as loading control.

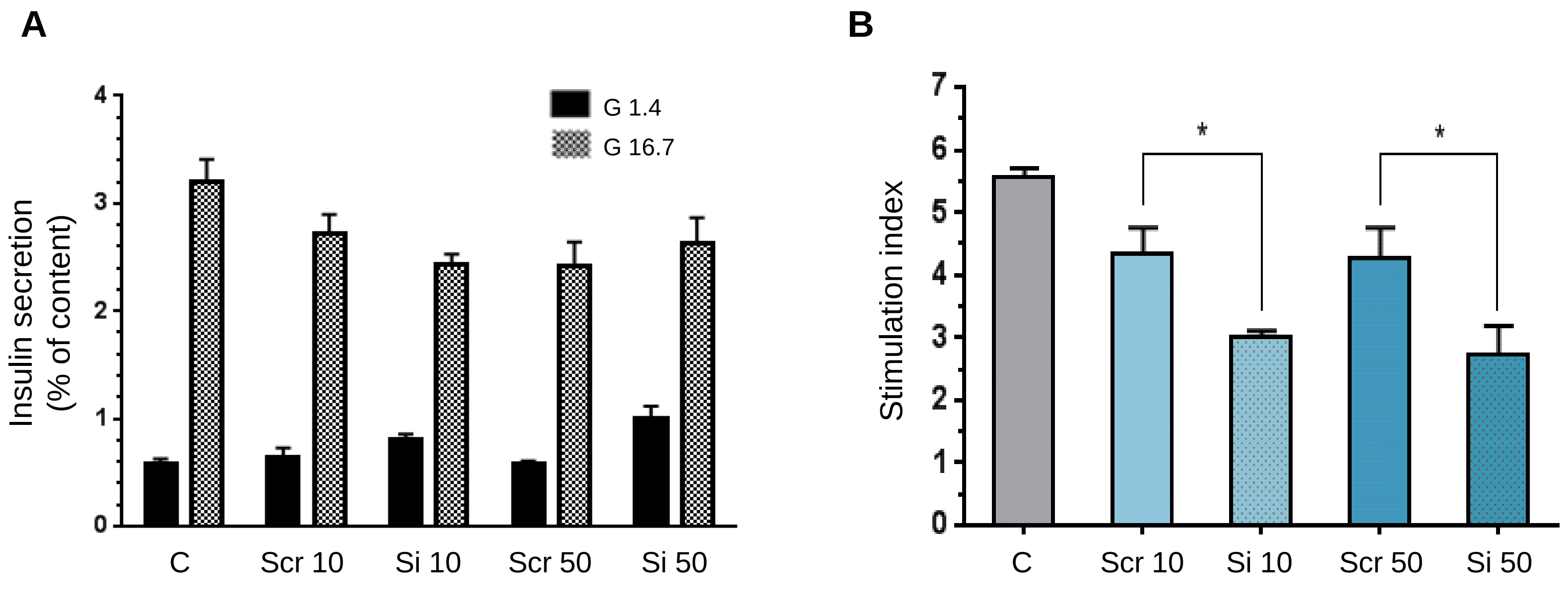


**Supplemental Figure 4. Knock-down of p300 by siRNA leads to altered glucose-induced insulin secretion in beta-cells. (A)** INS-1E cells were transfected with scramble (Scr) or p300 siRNA (Si) (10 or 50 nM) during 72h; (C, non-transfected cells). Following a 2-h quiescent period in Krebs 1.4 mM glucose (G1.4), cells were stimulated with 16.7 mM glucose (G16.7) during 1h at 37°C; (G1.4 refers to non-stimulated cells). Insulin secretion and insulin content were measured by HTRF (Homogeneous Time Resolved Fluorescence). The insulin content remained unchanged. The graph represents insulin secretion normalized to insulin content. (**B**) The graph represents the stimulation index (ratio G16.7: G1.4). Data are expressed as mean ± SEM (n=3); *P<0.05.

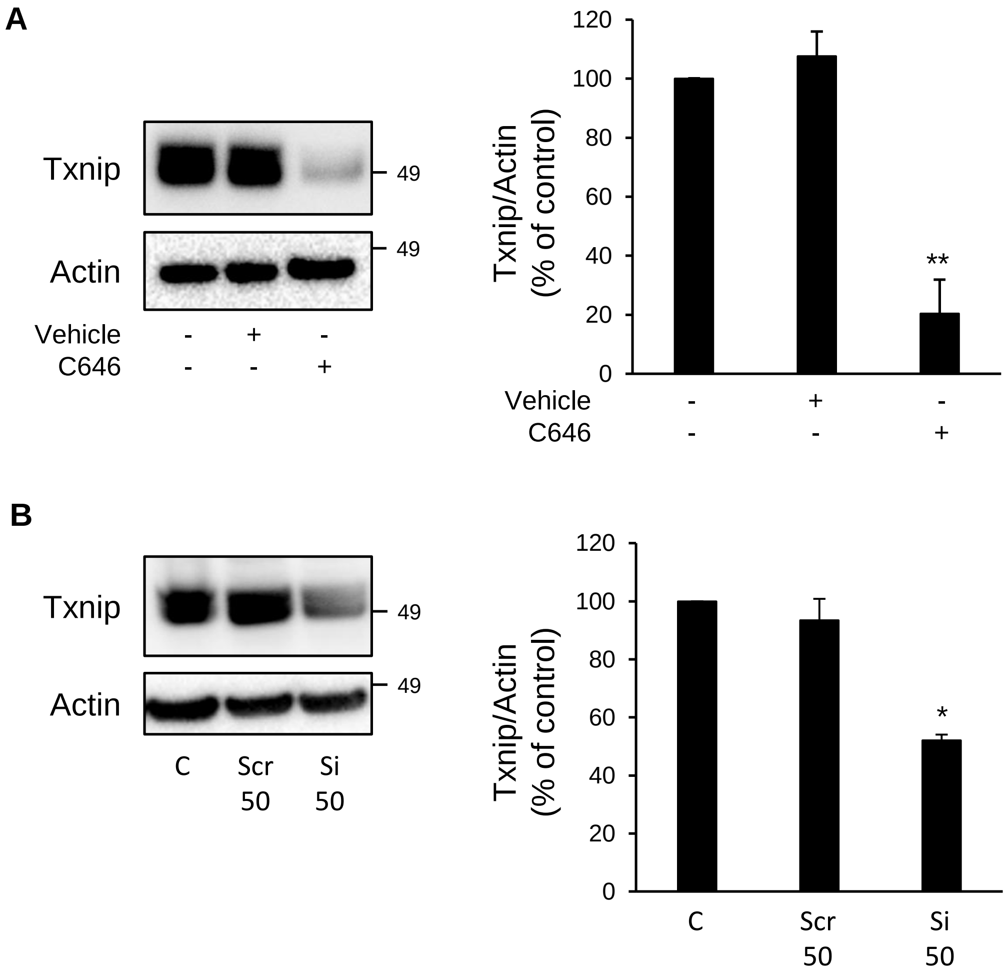


**Supplemental Figure 5. Txnip protein expression is decreased in INS-1E cells under inhibition or knock-down of p300. (A)** INS-1E cells were treated with C646 (30 μM for 24h) (or with 0.003% DMSO as vehicle). Levels of Txnip were assessed by western blot. Actin was used as loading control. The graph represents the quantification of the western blot (n=4). **(B)** INS-1E cells were transfected with scramble (Scr) or p300 siRNA (Si) (50 nM) during 48h; (C, non-transfected cells). Txnip protein levels were analyzed by western blot. Actin was used as loading control. The graphs represent the quantification of the western blot (n=3). Data are expressed as mean ± SEM; *P<0.05, **P<0.01.
