# Supplementary material for: Proteasomal degradation of the histone acetyl transferase p300 contributes to beta-cell injury in a diabetes environment
Source: Cell Death Dis. 2018 May 22;9(6):600. doi: 10.1038/s41419-018-0603-0 (PMC5964068; doi:10.1038/s41419-018-0603-0)
Supplement: Supplementary file 2 — Supplemental Materials and Methods [file 41419_2018_603_MOESM2_ESM.docx]

**SUPPLEMENTAL MATERIALS AND METHODS**

**TUNEL and immunostaining on isolated mouse islets and human islets.** Islets were isolated from four 4-month-old male mice and treated with C646 (30 μM, 72h) or DMSO as vehicle (130 islets per condition). Human islets obtained from 2 non-diabetic donors were treated according the same procedure. Islets were then washed twice in PBS and transferred into 3.7% paraformaldehyde (Sigma-Aldrich) in PBS for fixation during 4h. After 2 washes in PBS, islets were colored in 0.1% neutral red, washed twice in PBS and embedded in 24% agar. Cubes of agar containing batches of islets were fixed overnight into 3.7% paraformaldehyde, washed with PBS before being transferred into 70% ethanol and then processed for paraffin embedding. The percentage of apoptotic beta-cells (TUNEL-positive and insulin-positive cells) and apoptotic alpha-cells (TUNEL-positive and glucagon-positive cells) was determined on 4 µm-thick sections using the In Situ Cell Death Detection Kit - TMRred (Roche Diagnostics) following the manufacturer’s instructions. Sections were then incubated 1h at room temperature with primary antibodies (anti-insulin, 1/200, Abcam; anti-glucagon, 1/400, Sigma), followed by 1h at room temperature with secondary antibodies and DAPI. Sections were mounted in Mowiol (Sigma-Aldrich). Islets were imaged using a 20X objective from AxioImager microscope (Zeiss) using the Zen Blue software. Islets were imaged and saved in “.czi” (rather than “tiff”) format allowing to save the entire image parameters and were reopened and quantified using ImageJ (National Institutes of Health, Bethesda, MD). To quantify percentage of TUNEL positive beta-cells, 4000-6000 beta-cells per mouse were examined in detail and counted for presence/absence of TUNEL immunoreactivity. To quantify percentage of TUNEL positive alpha-cells, 600-1000 alpha-cells per mouse were examined for TUNEL immunoreactivity. To quantify percentage of TUNEL positive beta-cells and alpha-cells, 4500-6000 human beta-cells and 600-700 human alpha-cells were examined in detail and counted for presence/absence of TUNEL immunoreactivity.
